# Purification of Isosorbide via Ion Exchange Resin for High-Performance Bio-Based Polycarbonate

**DOI:** 10.3390/ma19010030

**Published:** 2025-12-21

**Authors:** Fei Han, Yan Zhang

**Affiliations:** Key Laboratory of Specially Functional Polymeric Materials and Related Technology (Ministry of Education), School of Materials Science and Engineering, East China University of Science and Technology, Shanghai 200237, China; y30220797@mail.ecust.edu.cn

**Keywords:** ion exchange resin, isosorbide, polycarbonate, bio-based, purification

## Abstract

Isosorbide-based polycarbonate (PIC) shows promising applications in medical devices and food packaging, owing to its excellent thermal stability, biosafety, optical properties, and scratch resistance. However, trace impurities, especially formic acid in isosorbide (ISB), can significantly influence the performance of PIC. Crude ISB was purified via ion exchange resin. Furthermore, the chromatographic parameters and the adsorption kinetics of formic acid were investigated. The results show that adsorption of formic acid follows the pseudo-second-order kinetic model, suggesting that it is chemisorption. The performance of PIC synthesized from purified ISB has been improved significantly, exhibiting number-average molecular weight (M_n_) of 53.9 kg/mol, elongation at break of 10.2%, and Young’s modulus of 1.84 GPa, along with notably enhanced thermal and optical properties.

## 1. Introduction

Polycarbonate (PC) is widely used in electronics, automotive components, and protective equipment because of its excellent thermal stability and mechanical strength [1,2,3,4]. However, traditional bisphenol A-based polycarbonate (BPA-PC) has been restricted in food packaging and medical devices due to the estrogenic effects of bisphenol A (BPA) [5,6,7]. Moreover, petroleum-derived BPA causes negative effects on the environment [8,9]. Therefore, research on biosafe and sustainable alternatives to BPA is highly significant.

Isosorbide (ISB), derived from renewable starch or cellulose, is non-toxic and biodegradable [10]. The two rigid and chiral tetrahydrofuran rings endow isosorbide-based polycarbonate (PIC) with superior thermal stability, optical properties, and scratch resistance [11,12,13,14,15,16,17]. However, the synthesis of high-performance PIC is still challenged by the reactivity difference between the exo-OH and endo-OH groups of ISB [18,19]. Many highly effective catalysts such as alkaline metals, alkaline earth metals, and ionic liquid catalysts have been developed to increase the molecular weight of the PIC [20,21,22]. Nevertheless, there are various impurities, including d-sorbitol, stereoisomers (isomannide and isoidide), mono-dehydration byproducts (1,4-sorbitan), and degradation products (formic acid, 2-acetylfuran) in the crude ISB [23,24,25]. The trace impurities, especially formic acid, could significantly reduce the activity of the basic catalysts, thus suppressing the increase in the PIC molecular weight. In addition, side reactions such as branching, crosslinking, and even gelation induced by impurities may further result in inferior mechanical and optical properties [26]. For example, the color of PIC in methylene chloride changes from light yellow to brownish yellow as the purity of ISB decreases from 99.8% to 98.0% [27]. The M_η_ of PIC is dramatically reduced from 28.2 kg/mol to 10.4 kg/mol and the T_g_ from 172.3 °C to 157.6 °C when the amount of impurity is increased from 0.5% to 5 mol% [25].

Many technologies have been explored for the purification of ISB. For instance, a thin-film evaporator was employed to purify ISB, and the purity of the final product reached 98.5% [28]. Nevertheless, the boiling point of ISB is as high as 372 °C. In order to purify ISB, the distillation temperature should be increased to 160 °C with a vacuum of just 0.60 kPa. Meanwhile, ISB is susceptible to reactions with trace oxygen at such high temperatures, leading to degradation and color deterioration. As reported by Hubbard, the purity of the ISB was raised to 99.35% through a mild recrystallization method but with a low yield of 60% [29].

The ion exchange resin can effectively remove impurities, especially formic acid, from ISB through an exchange reaction between its exchangeable OH^−^ ions and impurity ions [30,31,32,33]. Moreover, the resin is regenerable due to the reversible nature of the ion exchange reaction [34]. Compared to other methods, ion exchange chromatography is more efficient and selective. For example, the acetic acid and hydroxymethylfurfural can be totally removed from xylose by the A-103-S resin [35]. Meanwhile, this method operates under mild conditions, which allows ISB to remain stable at an ambient temperature. Furthermore, it delivers higher yield, lower energy consumption, and better economic efficiency.

Herein, crude ISB, which contained formic acid as the primary acidic impurity along with trace furan impurities, was purified via ion exchange chromatography. The chromatographic parameters, including solution flow rate and column temperature, were systematically evaluated through single-column experiments. To reveal the adsorption mechanisms and controlling steps, the adsorption kinetics of formic acid were further studied. The results indicate that film diffusion is the dominant control step, while chemical reaction is the second control step. Therefore, the selection of solution flow rate and column temperature are important for optimizing purification efficiency. Our study reveals that formic acid impurities in ISB significantly affect the properties of PIC. When the formic acid impurity content is high, it may lead to a decrease in the molecular weight of PIC and an increase in terminal -OH and -OPh groups content, thereby deteriorating the thermal and mechanical properties of PIC. Finally, the purified ISB with a pH of 7.57 was successfully obtained. The PIC synthesized from purified ISB exhibits a high molecular weight and excellent thermal, optical, and mechanical properties. It provides an effective method for the purification of crude ISB and high-performance PIC synthesis.

## 2. Materials and Methods

### 2.1. Materials

The crude ISB (purity: 98.85%) was provided by Ningbo Dafeng Jiangning New Materials Co., Ltd. (Ningbo, China). The chloride-form strong basic anion exchange resin, Amberlite IRA-402, was purchased from Aladdin (Shanghai, China), with a matrix of styrene–divinylbenzene copolymer and functional groups of -N^+^(CH_3_)_3_. The deionized water used in the experiments was purchased from Nongfu Spring Group Co., Ltd. (Hangzhou, China). Diphenyl carbonate (DPC, purity: 99%), hydrochloric acid (HCl, chemically pure), and sodium hydroxide (NaOH, chemically pure) were obtained from Sinopharm Chemical Reagent Co., Ltd. (Shanghai, China).

### 2.2. Ion Exchange Resin Pre-Treatment

To remove potential impurities, the ion exchange resin was pre-treated before use. It was first soaked in 10 wt% NaCl solution for 24 h and then in 4~5 wt% NaOH, 4~5 wt% HCl, and 4~5 wt% NaOH solution for 4 h, successively. The resin was rinsed thoroughly with sufficient deionized water after each immersion until the pH of the effluent became neutral.

### 2.3. Purify Isosorbide by Ion Exchange Chromatography

The purification efficiency of the ion exchange resin was systematically evaluated through single-column experiments. Experiments were conducted in downflow mode at flow rates of 1, 2, 3, 5, and 10 mL/min and temperatures of 25 °C, 35 °C, and 45 °C, respectively. The purified ISB is named ISB-RX and ISB-TY, where X and Y represent the specific values of flow rate and temperature, respectively.

### 2.4. Adsorption Kinetics Studies

The crude ISB was dissolved in a mixed solvent of deionized water and ethanol. The mass ratio of deionized water and ethanol was 1:1. The initial concentration of formic acid (C_0_) in this solution was 1.40 mg/L. For the adsorption kinetics studies, 80 mL of the crude ISB solution and 12 g of ion exchange resin were added together into a conical flask and stirred at 25 °C. The formic acid content was analyzed at regular intervals. The obtained data were fitted using pseudo-first-order kinetic and pseudo-second-order kinetic models and moving boundary model, respectively.

### 2.5. Synthetic Isosorbide-Based Polycarbonate (PIC)

**Figure 1:**
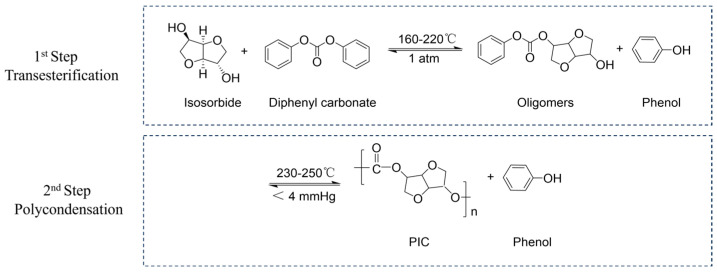
The synthesis of isosorbide-based polycarbonate via melt transesterification-polycondensation method.

PIC was synthesized by the melt transesterification-polycondensation method as the reported method [22]. The molar ratio of DPC to ISB was maintained at 1.01:1 using Cs_2_CO_3_ as the catalyst, with its amount set at 0.16 mol% relative to ISB. The monomers and catalyst were heated to 160 °C under a nitrogen atmosphere to make the reagents fuse completely. After stirring for 20 min, the temperature was gradually increased to 180 °C and maintained for 30 min to drive the transesterification, followed by a further increase to 220 °C for 10 min. The total reaction time in the transesterification stage was about 90 min. Subsequently, the polycondensation stage was initiated by setting the temperature to 230 °C under vacuum (200 mmHg). After 10 min, the temperature was increased to 250 °C, while the degree of vacuum was further increased to 40 mmHg. After further reaction for 10 min, the degree of the system vacuum was increased to less than 4 mmHg, and the reaction was completed within 10 min. The total reaction time in the polycondensation stage was about 60 min. The synthesis reaction equations of isosorbide-based polycarbonate are illustrated in Figure 1.

### 2.6. Characterization

The pH was measured at room temperature by a pH meter (PHS-3CU, Yueping, Shanghai, China). The formic acid content in ISB was detected by an ion chromatograph (ICS-5000+, Thermo, Waltham, MA, USA). The chemical structure of ISB was characterized by Fourier transform infrared spectrometer (Nicolet 5700, Thermal Fisher, Waltham, MA, USA). The number-average molecular weight (M_n_), weight-average molecular weight (M_w_), and polydispersity index (PDI) were measured by GPC (1260 Infinity II, Santa Clara, CA, USA). N, N-dimethylformamide (DMF) was used as an eluent with a flow rate of 1.0 mL/min at 40 °C. Polystyrene was used as the standard. The ^1^H NMR and ^13^C NMR spectra of PIC were obtained by an NMR spectrometer (Avance-400 MHz, Bruker, Billerica, MA, USA). Deuterated chloroform (CDCl_3_) was used as an analytical solvent, and chemical shift values (δ) were determined in ppm with reference to internal tetramethylsilane (TMS). The glass transition temperature (T_g_) was measured by DSC (Discovery 25, TA Instrument, New Castle, DE, USA). The sample was first heated from 20 to 220 °C at a rate of 30 °C/min, stabilized for 2 min to eliminate the thermal history, and then dropped to 20 °C at the same rate. Finally, the temperature was raised to 250 °C at a rate of 10 °C/min. T_g_ was determined as the inflection point of the second heating curve. Thermal stability was evaluated from 30 °C to 800 °C at a rate of 10 °C/min under a nitrogen atmosphere by thermogravimetric analysis (STA 449C, NETZSCH, Selb, Germany). X-ray diffraction (XRD) analysis was performed using a diffractometer (D8 Advance, Bruker, Billerica, MA, USA) with Cu- Kα radiation at 30 kV and 30 mA. The diffraction patterns were recorded in the 2θ range of 5° to 50° at a scanning rate of 5°/min. The visible light transmittance of the PIC film with a thickness of 100 µm was tested by UV spectrophotometer (UV 1900, Youke, Shanghai, China) in the range of 200−900 nm. The stress–strain tests of PIC were measured with a universal testing machine (Instron 3365, Instron Corporation, Norwood, MA, USA) at a speed of 10 mm/min. The measurement was repeated five times to obtain an average value. The morphology of the cryo-fractured surfaces of the PIC samples was observed on scanning electron microscope (SEM, Hitachi S-3400, Tokyo, Japan). MS analysis was conducted by MALDI-TOF-MS (Voyager-DE STR, Applied Biosystems, Foster City, CA, USA).

## 3. Results and Discussion

### 3.1. FTIR Spectra and HPLC Chromatogram of ISB Before and After Purification

**Figure 2:**
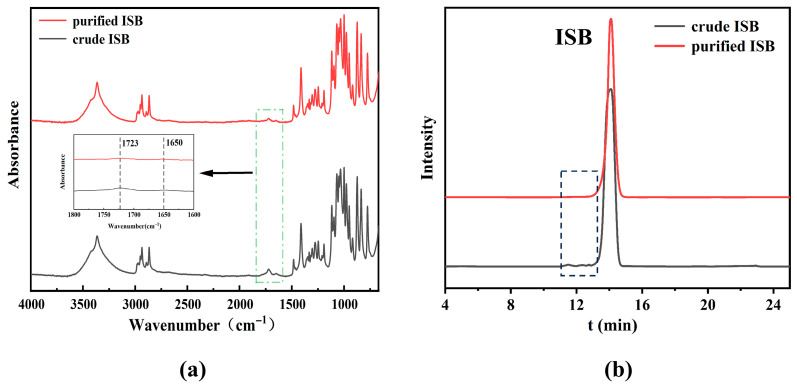
(**a**) FTIR spectra and (**b**) HPLC chromatogram of crude ISB and purified ISB.

FTIR spectra of ISB before and after purification are displayed in Figure 2a. The absorption peaks at 1480 cm^−1^ and 1390 cm^−1^ are assigned to C-H bending vibrations, while the peaks at 1040 cm^−1^ and 1000 cm^−1^ are attributed to C-O and C-O-C stretching vibrations, respectively. Notably, weak absorption peaks are detected at 1723 cm^−1^ and 1650 cm^−1^. The former originates from C=O stretching vibrations in residual aldehyde-containing compounds such as formic acid and furan, while the latter may result from conjugated ketone carbonyl (C=O) vibrations generated through aldol condensation reactions of furfural-type impurities. Given that the crude ISB already possesses high purity (>98%), the intensities of both peaks are tiny. The O-H stretching vibration of ISB at 3360 cm^−1^ is used as an internal standard to calculate the relative peak areas at 1723 cm^−1^ and 1650 cm^−1^. After purification, the relative peak areas at 1723 cm^−1^ and 1650 cm^−1^ decreased from 0.065 and 0.043 to 0.026 and 0.018, respectively, demonstrating the effective removal of formic acid and furan impurities from ISB.

HPLC analysis was performed for the crude and purified ISB, and the results are shown in Figure 2b. It can be seen that the crude ISB shows weak impurity peaks at 11–13 min, which are essentially eliminated after purification. The main peak of ISB appears at 14.1 min. Based on the area normalization method, the purity of ISB improved from 98.80% before purification to 99.52% after purification. These results clearly demonstrate that the ion exchange resin used in this study is highly effective for purifying ISB.

### 3.2. The Influence of Chromatographic Parameters on Purification Efficiency

**Figure 3:**
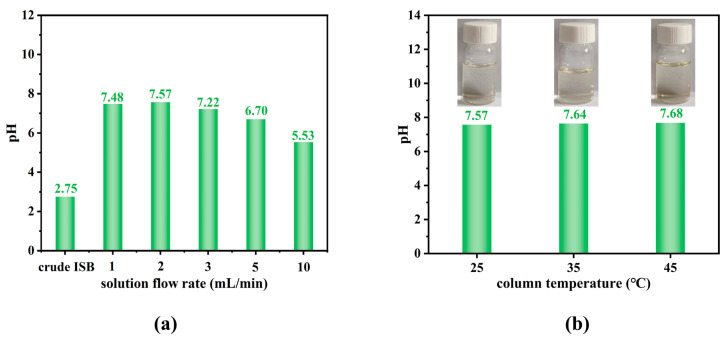
The influence of chromatographic parameters on purification efficiency: (**a**) solution flow rate, (**b**) column temperature.

The formic acid impurities first diffuse through the liquid film to the resin surface and are then removed through exchange between HCOO^−^ ions and OH^−^ ions of the resin. The contact time between the ion exchange resin and formic acid impurities depends on the flow rate of the solution. Therefore, the effect of the flow rate of the ISB solution on the removal efficiency of formic acid impurities is further investigated. Considering the processing efficiency and the final purification effect, the selected flow rates are 1, 2, 3, 5, and 10 mL/min, and the results are displayed in Figure 3a.

According to the Prandtl boundary layer theory, δ is proportional to U^−0.5^, where δ is the boundary layer thickness and U is the flow rate, respectively [36]. Therefore, the boundary layer thickens with the decrease in the solution flow rate, indicating that the formic acid impurities diffuse through a thicker liquid film to the resin surface. While the lower flow rate increases contact time, it also leads to increased diffusion resistance and reduced mass transfer rates, ultimately resulting in a diminished purification efficiency at 1 mL/min. At a flow rate of 2 mL/min, the thickness of the liquid film is moderate, allowing sufficient time for exchange between HCOO^−^ ions and OH^−^ ions, resulting in a pH increase from 2.75 to 7.57. Further acceleration of ISB flow causes a significant decrease in purification efficiency. It seems the ion exchange resin has lost effective purification capability at 5 and 10 mL/min. The ISB solution flows so quickly that the formic acid impurities hardly have time to penetrate through the liquid film before elution, leading to a much lower pH value. Especially when the flow rate is 10 mL/min, the pH is only 5.53.

The operational temperature range of ion exchange resin is constrained by deactivation above 50 °C and ISB crystallization below 0 °C, prompting the selection of 25 °C, 35 °C, and 45 °C to systematically evaluate temperature effects on purification performance within relevant conditions. The changes in ISB pH value and color with the column temperature are presented in Figure 3b. It can be seen that there is little pH variation in the purified ISB. The exchange frequency between HCOO^−^ ions and exchangeable OH^−^ ions on the resin is enhanced with the temperature increases. Consequently, the separation efficiency for formic acid impurities is improved slightly. However, the oxidative degradation of ISB may occur at a higher temperature, leading to discoloration.

### 3.3. Adsorption Kinetics

The adsorption kinetics of formic acid impurities were investigated to understand the adsorption mechanism and identify the controlling step in mass transfer. The adsorption capacity (q_t_, mg/g) for formic acid impurity can be calculated by Equation (1):

(1) $$q_{t}=\frac{\left(C_{0}-C_{t}\right)V}{m}$$

where C_0_ and C_t_ are the formic acid concentration (mg/L) in the crude ISB and purified ISB taken at time t, respectively. V is the volume of the solution (mL), and m is the amount of ion exchange resin used (g). q_t_ is the adsorption capacity of the ion exchange resin for formic acid at different times (mg/g).

The equilibrium adsorption capacity, q_e_ (mg/g), of the resin for formic acid is calculated according to Equation (2). C_0_ and C_e_ are 1.40 mg/L and 0.46 mg/L, respectively. The q_e_ calculated is 6.27 mg/g:

(2) $$q_{e}=\frac{\left(C_{0}-C_{e}\right)V}{m}$$

where C_e_ is the equilibrium concentration (mg/L) of formic acid.

The adsorption process of formic acid was fitted using pseudo-first-order and pseudo-second-order kinetic models, respectively. The pseudo-first-order kinetic model corresponds to physical adsorption, and the fitting equation is as follows:

(3) $$q_{t}=q_{e}\left(1-e^{-k_{1}t}\right)$$

where k_1_ is the rate constant (min^−1^) of the pseudo-first-order kinetic model.

The pseudo-second-order kinetic model corresponds to chemical adsorption, and the fitting equation is as follows:

(4) $$q_{t}=\frac{k_{2}q_{e}^{2}t}{1+k_{2}q_{e}t}$$

where k_2_ is the rate constant (g/mg/min) of the pseudo-second-order kinetic model.

**Figure 4:**
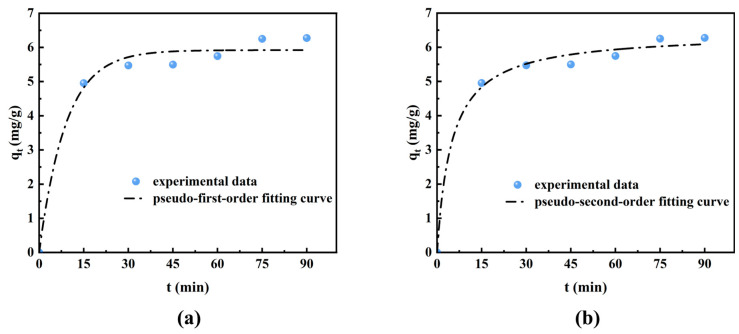
Kinetic fitting curves of formic acid adsorption by ion exchange resin: (**a**) pseudo-first-order model, (**b**) pseudo-second-order model.

The corresponding fitting results are shown in Figure 4 and Table 1.

An initial rapid increase in adsorption capacity is observed within the first 15 min, which is attributed to the abundant unoccupied adsorption sites and high concentration of formic acid concentration in the ISB solution. The adsorption rate then decreases as the formic acid concentration decreases and electrostatic repulsion emerges [37] until the adsorption equilibrium is reached. The correlation coefficient (R^2^) of the pseudo-second-order kinetic model is 0.992, much higher than that of the pseudo-first-order kinetic model. Moreover, the equilibrium adsorption capacity (q_e_) predicted by the pseudo-second-order model is 6.42 mg/g, which is much closer to the experimental value of 6.27 mg/g. The results suggest that the adsorption of formic acid by the ion exchange resin follows the pseudo-second-order kinetic model, which is a chemical adsorption process and the HCOO^−^ forms ionic bonds with the -N^+^(CH_3_)_3_ functional groups of the ion exchange resin [38]. The adsorption capacity depends on the -N^+^(CH_3_)_3_ functional group density of the ion exchange resin, thus lower crosslinking degrees of the ion exchange resin are preferred. In addition, the adsorption capability of the ion exchange resin can be restored by using strong alkaline solutions to cleave the ionic bonds when the formic acid adsorption reaches saturation.

### 3.4. Moving Boundary Model

**Figure 5:**
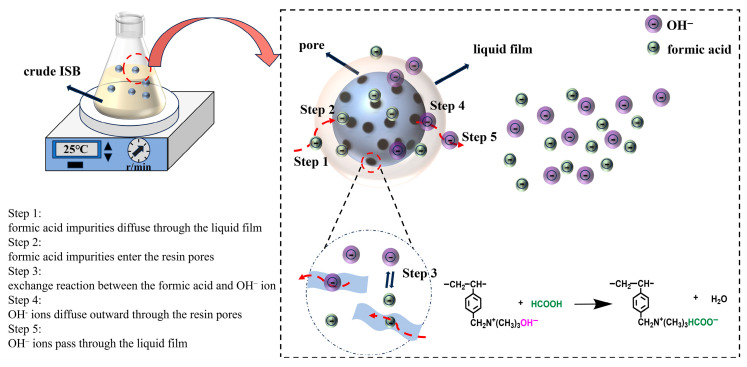
Schematic diagram of the ion exchange process.

The adsorption of formic acid impurities in ISB by the ion exchange resin is a chemical adsorption process. However, it is very complex, usually including the following five steps. At first, the formic acid impurities in the solution diffuse through the liquid film surrounding the ion exchange resin particles. Then, the formic acid impurities enter the internal pores of the resin and migrate toward active sites. The formic acid impurities react with exchangeable OH^−^ ions. The displaced OH^−^ ions diffuse outward through the resin pores. Finally, OH^−^ ions pass through the liquid film and enter the solution. From a mass transfer mechanism perspective, steps 1 and 5 are film diffusion, steps 2 and 4 belong to particle diffusion, while step 3 corresponds to the chemical reaction. The adsorption rate is determined by the slowest step. The adsorption process of formic acid and the corresponding exchange reaction are illustrated in Figure 5.

The moving boundary model, which accurately describes mass transfer characteristics, is widely used to analyze the adsorption behavior of ion exchange resin. The kinetic equations of film diffusion, particle diffusion, and chemical reaction are shown in Equations (5)–(7) [39].

Film diffusion:

(5) $$F=-k_{1}t$$

Particle diffusion:

(6) $$1-3{\left(1-F\right)}^{\frac{2}{3}}+2\left(1-F\right)=k_{2}t$$

Chemical reaction:

(7) $$1-{\left(1-F\right)}^{\frac{1}{3}}=k_{3}t$$

where F is the reaction extent, F = q_t_/q_e_. k_1_ and k_2_ are the film and particle diffusion coefficient (min^−1^), respectively. k_3_ is chemical reaction rate constant (min^−1^).

**Figure 6:**
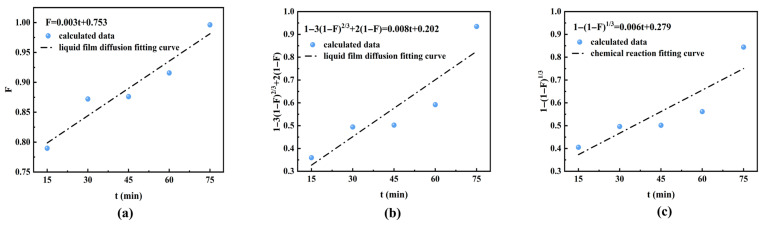
(**a**) Film diffusion, (**b**) particle diffusion, and (**c**) chemical reaction control fitting curves.

Based on the moving boundary model, the adsorption process of formic acid impurities by ion exchange resin is further analyzed. The fitting results are shown in Figure 6, and the related data are listed in Table 2.

The diffusion coefficient k_1_ of film diffusion and k_3_ of chemical reaction are both 0.006, lower than k_2_ of particle diffusion. The similar values of k_1_ and k_3_ indicate a balanced matching between film diffusion and chemical reaction, with neither serving as an absolute rate controlling step, reflecting an inherent coordination at the kinetic level of the system. At the same time, the adsorption process exhibits dynamic equilibrium characteristics, making it difficult to linearly enhance the overall efficiency by solely strengthening any single aspect. However, the R^2^ of liquid film diffusion control is 0.926, which is much higher than the others. It indicates that the rate of the entire adsorption process is determined by both the diffusion of formic acid impurities through the liquid film and the diffusion of OH^−^ ions through the liquid film. The fitting results agree well with the experimental data, as the pH of purified ISB is as high as 7.57 at the flow rate of 2 mL/min. Furthermore, the chemical exchange reaction between the HCOO^−^ of formic acid and OH^−^ on the functional groups of the resin is the second control step. According to the moving boundary model analysis, the primary optimization measure should focus on improving hydrodynamic conditions. This can be achieved by appropriately increasing the flow rate in column operations to reduce the thickness of the liquid film boundary layer. On this basis, moderately raising the temperature can further enhance the purification efficiency of ISB.

### 3.5. Synthesis of High-Performance PIC

#### 3.5.1. Molecular Weight and Chemical Structure

**Figure 7:**
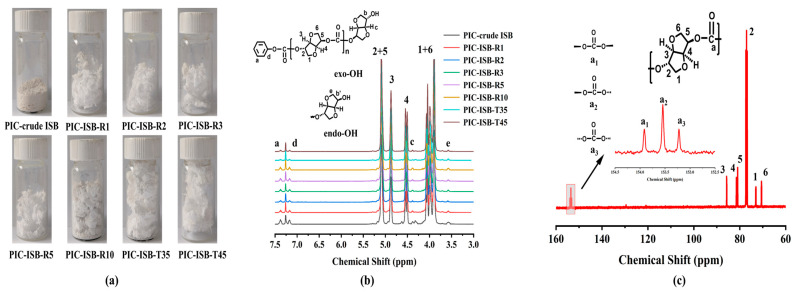
(**a**) The photographs and (**b**) the ^1^H NMR spectra of PIC synthesized from ISB with different properties; (**c**) the ^1^C NMR spectra of PIC-ISB-T45. Here, PIC-crude ISB is the PIC synthesized from crude ISB. PIC-ISB-R1, PIC-ISB-R2, PIC-ISB-R3, PIC-ISB-R5, and PIC-ISB-R10 are the PIC synthesized from ISB purified at flow rates of 1, 2, 3, 5, and 10 mL/min, respectively. PIC-ISB-T35 and PIC-ISB-T45 are the PIC synthesized from ISB purified at 35 °C and 45 °C, respectively.

PIC was synthesized from ISB with different properties, and the photographs of the synthesized PIC samples are shown in Figure 7a. The PIC synthesized from crude ISB is powdery and brownish yellow in color, whereas the PIC synthesized from purified ISB is flocculent. As the pH of the ISB increases, the color of PIC changes from light yellow to white. The number-average molecular weight (M_n_), weight-average molecular weight (M_w_), and polydispersity index (PDI) of the obtained PIC are listed in Table 3.

**Figure 8:**
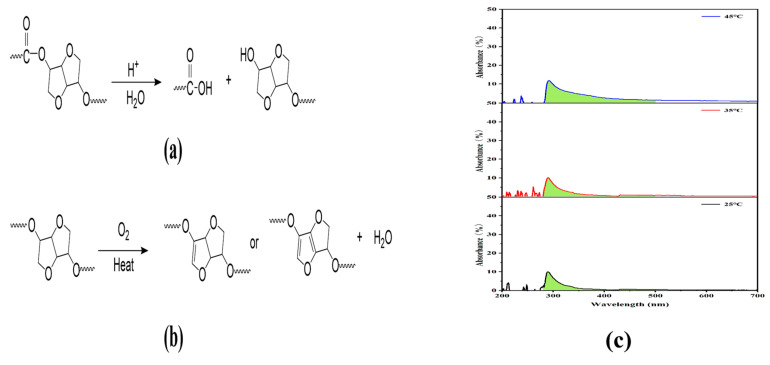
(**a**) The proposed mechanism for the acid-catalyzed hydrolysis of PIC, (**b**) possible chromophoric furan structures in PIC chains, and (**c**) UV-Vis absorption spectra of purified ISB.

As the pH of ISB is increased from 2.75 to 7.68, the M_n_ of the synthesized PIC rises from 10.1 kg/mol to 53.9 kg/mol, and the M_w_ increases from 17.9 kg/mol to 85.2 kg/mol, respectively. The increase in molecular weight is mainly attributed to the effective removal of acidic impurities and trace furan impurities from crude ISB. Among these, the removal of acidic impurities represented by formic acid is particularly crucial, as the acidic impurities in ISB may reduce the activity of basic catalysts, thereby suppressing the transesterification of DPC and ISB. Simultaneously, the acidic impurities may further catalyze the hydrolysis of ester bonds in PIC chains [40]. The PIC chains are interrupted by hydrolysis, leading to a decrease in molecular weight and an increased PDI, as illustrated in Figure 8a.

The chemical structure of the PIC synthesized from ISB with different properties was characterized by ^1^H NMR and ^13^C NMR spectroscopies, as displayed in Figure 7b,c, respectively. All proton signals are observed at the expected positions. In the repeating units, the peaks of the ISB moiety at δ 4.08, 5.11, 4.89, 4.54, 5.09, and 3.89 ppm are assigned to H-1, H-2, H-3, H-4, H-5, and H-6, respectively. The peaks of the terminal groups, including the ISB moiety and the DPC moiety, at 4.39, 3.58, and 7.38 ppm are assigned to the hydrogen atoms c, e, and a, respectively. Hydrogen atom c refers to the ISB containing a terminal exo-OH, while hydrogen atom e refers to a terminal endo-OH. The contents of terminal groups are calculated based on peak 3 from ISB. The peak at δ 4.89 ppm (peak 3) is normalized to 1, and the detailed data are listed in Table 3. It is noted that the terminal endo-OH content is higher than that of exo-OH in the PIC, which demonstrates that there is a lower reactivity of the endo-OH group in ISB during polymerization. After removing acidic impurities, the catalytic hydrolysis of PIC molecular chains is effectively suppressed, yielding PIC with higher molecular weights and more intact chain structures. Due to the reduced number of polymer chains per unit mass and more controllable chain-end structures, the overall content of terminal -OH and -OPh groups decreases significantly from 0.44 and 0.53 to 0.12 and 0.20, respectively.

The signals in the ^13^C NMR spectrum of PIC-ISB-T45 are in good correspondence with those of carbons in the PIC molecular chain. The peaks of the ISB moiety in the repeating unit at δ70.6, 72.8, 76.9, 85.7, 80.9, and 70.5 ppm are assigned to C-1, C-2, C-3, C-4, C-5, and C-6, respectively. The peak of the DPC moiety at δ153.0–154.0 ppm is assigned to carbon atom a, including a_1_ at δ153.9 ppm, a_2_ at δ153.5 ppm, and a_3_ at δ153.2 ppm. The peak area of a_2_ is normalized to 1, and the peak areas of a_1_ and a_3_ of all the PIC samples are calculated, with the results listed in Table 3. The a_1_: a_2_: a_3_ ratio of all PIC samples is close to 1:2:1, which indicates the random sequence configuration of PIC from a statistical view [17].

#### 3.5.2. Thermal Properties

**Figure 9:**
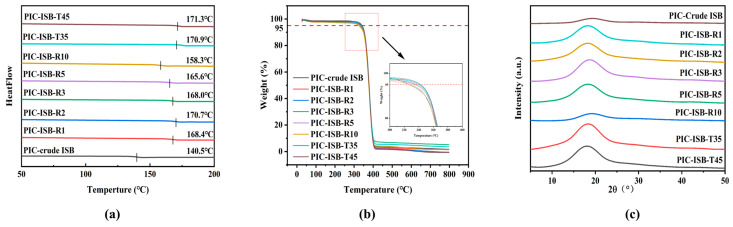
(**a**) DSC, (**b**) TGA, and (**c**) XRD curves of PIC synthesized from ISB with different properties.

The thermal properties are highly important for the processing and application of the materials. The rigid furan ring of ISB endows PIC with superior thermal performance. However, the trace formic acid impurities may greatly lower the activity of the basic catalysts, thus influencing the performance of the PIC. The thermal properties of PIC were further analyzed by DSC and TGA, and the curves are shown in Figure 9a,b. There is no obvious crystallization or melting peak in the DSC curve of PIC, indicating it is completely amorphous. To further study the amorphous nature of PIC, X-ray diffraction (XRD) was performed. Only a broad halo peak around 2θ ≈ 18° is observed in Figure 9c, with no sharp crystalline peaks, confirming their fully amorphous nature. The intensity of the diffuse XRD peaks varies among the PIC samples, which may arise from differences in molecular weight and chain entanglement density. Specifically, samples with a higher molecular weight (e.g., PIC-ISB-T45) exhibit stronger scattering intensity, consistent with a denser packing of polymer chains in the amorphous phase. After the crude ISB is purified, the glass transition temperature (T_g_) of PIC increases from 140.5 to 171.3 °C. The T_g_ of PIC is mainly determined by the molecular weight and molecular structure. Higher molecular weight results in longer polymer chains and increased chain entanglement, which imposes greater constraints on segmental motion and consequently elevates the T_g_. Furthermore, the endo–endo (a_1_) structure is reported to be conducive to an increase in the T_g_ value. The motion of the a_1_ structure in the PIC chain is more restricted than that of the a_3_ structure during the transition from the glassy to the rubbery state, which leads to an increase in the T_g_ of the polymer [20]. As shown in Table 3, the removal of impurities leads to an increase in the a_1_/a_3_ ratio, which contributes to the enhancement of the T_g_. Notably, PIC synthesized from ISB-T45 exhibits the highest T_g_ of 171.3 °C, suggesting that it has excellent thermal properties.

Furthermore, PIC synthesized from purified ISB displays good thermal stability. There is no obvious weight loss before 300 °C, owing to adjacent furan rings in the PIC backbone. As the pH of ISB is increased from 2.75 to 7.68, the T_d-5%_ of PIC increases from 326.9 to 341.7 °C. However, when the pH of ISB exceeds 7.5, further enhancement in the T_d-5%_ of PIC becomes slight. PIC with lower terminal -OH group contents exhibit superior thermal stability because terminal -OH groups are thermally unstable functional groups that can initiate degradation [22]. Wang et al. employ ISB with 98% purity, which is further purified by triple recrystallization in acetone, yet they obtain a PIC product with a T_g_ of only 169 °C and a T_d-5%_ of 310 °C [20]. In comparison, the PIC-ISB-T45 synthesized in this study exhibits a higher T_g_ of 171 °C and a T_d-5%_ of 341 °C. Notably, this T_g_ value even exceeds that of a PIC sample synthesized from ISB with a purity as high as 99.8% (T_g_ = 170.3 °C) [41]. PIC exhibits a significantly higher T_g_ than BPA-PC (150 °C), which is attributed to the strong restriction of chain mobility imposed by the rigid furan ring structure in ISB, thereby endowing PIC with superior resistance to thermal deformation. However, the T_d-5%_ of PIC is lower than that of BPA-PC (410 °C), primarily due to the possible dehydration and ring-opening reactions of ISB at elevated temperatures. Its thermal degradation mechanism thus differs from that of aromatic ring-based BPA-PC.

#### 3.5.3. Optical Properties

**Figure 10:**
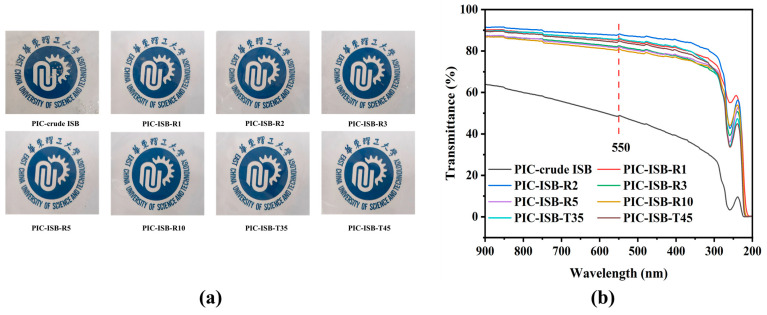
(**a**) The photos and (**b**) visible light transmission of PIC films.

Optical properties are very important for the application in the packaging and automotive industries. As displayed in Figure 10a, all the PIC films from purified ISB are transparent, whereas the PIC-crude ISB film is slightly yellowish. The transmittance curves of PIC films in the visible light region are displayed in Figure 10b, and the related data are listed in Table 4. The PIC films synthesized from purified ISB exhibit good optical properties, with transmittance at 550 nm more than 80%. Notably, the transmittance of the PIC-ISB-R2 film is raised to 88.1%, while that of PIC-crude ISB is just 48.7%. BPA-PC exhibits a visible-light transmittance of approximately 85%, while the optical transmittance of PIC surpasses that of BPA-PC due to the rigid furan ring structure in ISB. The acidic impurities in crude ISB may catalyze ring-opening side reactions during the melt polycondensation, which further triggers cross-linking or elimination side reactions at high temperature to form gels or unsaturated chain ends. A series of side reactions ultimately leads to discoloration and the decreased transmittance of PIC films [42]. Although PICs from ISB-T35 and ISB-T45 have higher molecular weights and better thermal properties, the oxidative degradation of ISB may occur at higher temperatures, generating chromophoric furan structures in PIC chains, as shown in Figure 8b [43]. UV-Vis spectroscopy was performed on ISB samples purified at 25 °C, 35 °C, and 45 °C, and the results are shown in Figure 8c. Chromophoric furan structures can impair the optical transparency of PIC films, with their primary absorption occurring in the wavelength range of 280–500 nm. The absorption areas in this range for samples purified at 25 °C, 35 °C, and 45 °C are 6.7, 8.9, and 10.1, respectively. These results indicate that an increased purification temperature may intensify oxidative degradation, generating more chromophoric furan structures, which consequently affect the optical properties of PIC. Consequently, the transmittance of PIC-ISB-T35 and PIC-ISB-T45 films is less than that of the PIC-ISB-R2 film.

#### 3.5.4. Mechanical Properties

**Figure 11:**
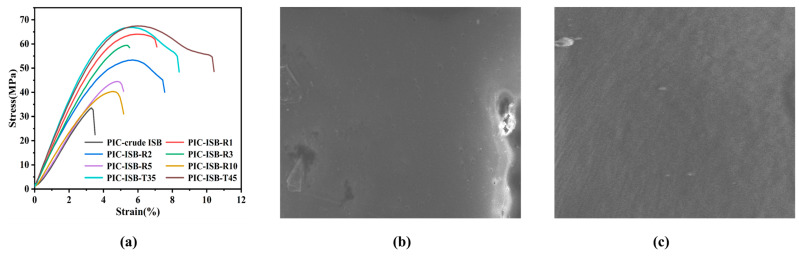
(**a**) Stress−strain curves of PIC synthesized from ISB with different properties, SEM images of the cryo-fracture surface of (**b**) PIC-crude ISB and (**c**) PIC-ISB-T45.

The stress–strain curves of PIC are displayed in Figure 11a, and the elongation at break and Young’s modulus are listed in Table 4. The Young’s modulus of PIC-crude ISB is only 1.11 GPa, which is mainly attributed to the insufficient chain entanglement density caused by its relatively low molecular weight. Under external stress, the polymer chains readily undergo slippage, leading to reduced rigidity. After the crude ISB is purified, the Young’s modulus of PIC-ISB-T45 is increased to 1.84 GPa. PIC generally shows brittle behavior due to the high rigidity of ISB. As the formic acid content decreases, the elongation at break of PIC only increases from 3.5% to 10.2%. Low toughness is an inherent drawback of ISB homopolycarbonate, so simply purifying ISB can hardly eliminate its brittleness. Introducing flexible segments such as cyclohexanedimethanol (CHDM) to copolymerize with ISB decreases the rigidity of PIC, thereby effectively enhancing the elongation at break [44]. The Young’s modulus of BPA-PC is approximately 2.0 GPa, while that of PIC is slightly lower. Nevertheless, the Young’s modulus of both materials falls within the same order of magnitude, indicating that PIC also possesses the fundamental rigidity required for engineering plastics. However, there is a significant difference in the elongation at break between PIC and BPA-PC (100–150%). The flexible isopropyl linkage in BPA-PC contributes to its outstanding toughness, whereas the rigid ISB units in PIC severely restrict the extensibility and plastic deformation capacity of the polymer chains, leading to brittle fracture behavior.

The SEM images of the cryo-fracture surface of the PIC samples are shown in Figure 11. As the testing temperature is well below the T_g_ of PIC, both samples exhibit a typical low-temperature brittle fracture mechanism. Nevertheless, the SEM images show significant differences, which are related to the molecular weight, chain entanglement, and structural defects of PIC. PIC-ISB-T45 has a higher molecular weight, corresponding to a greater chain entanglement density, which facilitates the formation of an effective entanglement network. Here, entanglement points act as physical cross-links that can induce crack deflection and branching during propagation, resulting in a rougher fracture morphology, as shown in Figure 11c. Conversely, PIC-crude ISB has a lower molecular weight and the associated weaker chain entanglement, leading to a flat, smooth fracture surface. Furthermore, acidic impurities and their volatile derivatives can introduce structural defects such as pores during the processing of PIC, which are also reflected in Figure 11b.

#### 3.5.5. Polymer Backbone Structure and Terminal Groups

**Figure 12:**
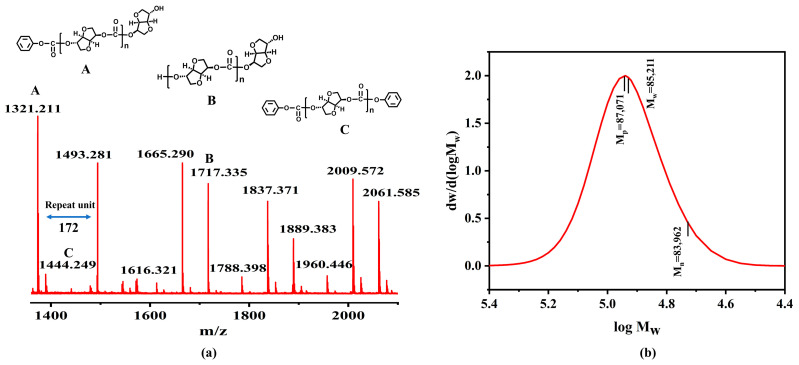
(**a**) MALDI-TOF-MS spectrum and (**b**) GPC curves of PIC-ISB-T45.

Several types of polymer terminal groups can result from the reaction between ISB and DPC; we used MALDI-TOF-MS to further investigate the polymer backbone structure and terminal groups. As displayed in Figure 12, some possible structures are detected; the monosubstituted and asymmetric substituted structures are detected as A, B, and C. The mass difference between peaks (*m*/*z* 172.5 ± 1.3) is consistent with the average mass of the PIC repeating units (172.14 g mol^−1^). The synthesized PIC-ISB-T45 is mainly composed of structure A, while the formation of structures B and C may be attributed to insufficient conversion caused by the increased viscosity of the reaction mixture [21].

The molecular weight and its distribution of PIC-ISB-T45 were determined by GPC, as shown in Figure 12b. It exhibits a single peak with a relatively symmetrical shape, indicating that the sample is primarily composed of a single polymeric component, with no detectable significant low- or high-molecular-weight impurities.

#### 3.5.6. Structure of the Transurethanization/Polycondensation Distillates

**Figure 13:**
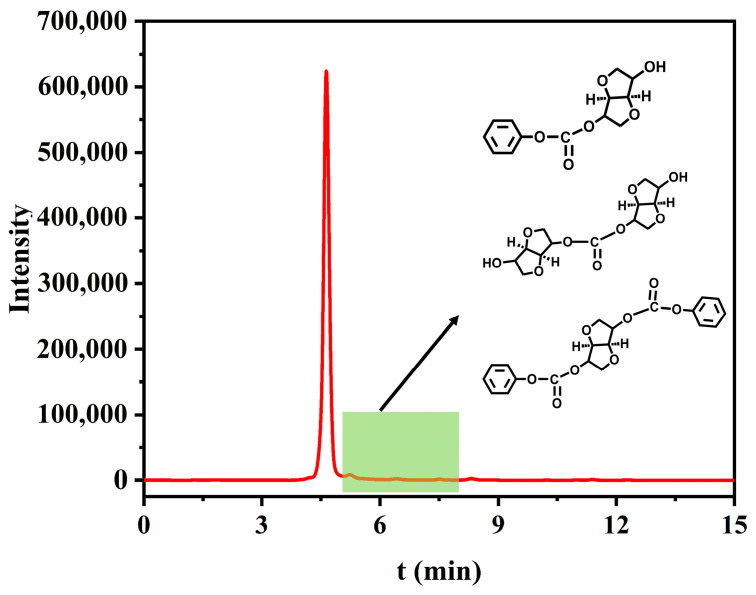
HPLC chromatogram of the transurethanization/polycondensation distillates.

The distillates from the transurethanization/polycondensation reaction were analyzed by high-performance liquid chromatography (HPLC) [45], with results shown in Figure 13. Based on retention times, the peak at 5.0 min was identified as the byproduct phenol, while the peak at 8.5 min corresponded to unreacted DPC. Minor low-intensity peaks appearing at other retention times were attributed to oligomers formed during the reaction. Potential structures [22] of these oligomers are proposed in Figure 13. Determined by area normalization, the relative peak areas of phenol, DPC, and oligomers are 95.28%, 0.53%, and 4.19%, respectively.

## 4. Conclusions

Crude ISB was purified via ion exchange resin. The purified ISB with a pH of 7.68 and formic acid content of 0.34 mg/L was obtained by optimizing chromatographic parameters. The adsorption of formic acid follows the pseudo-second-order kinetic model, suggesting that it is chemisorption. Moreover, the adsorption process is controlled by both film diffusion and chemical reaction. Analysis of the chemical structure of PIC confirms that after the removal of impurities, the content of terminal -OH and -OPh groups decreases from 0.44 and 0.53 to 0.12 and 0.20, respectively. This reduction contributes to the enhanced thermal properties. Compared to PIC from crude ISB, PIC synthesized from purified ISB exhibits excellent performance, with a number-average molecular weight (M_n_) of 53.9 kg/mol, light transmittance at 550 nm of 84.7%, elongation at break of 10.2%, Young’s modulus of 1.84 GPa, and T_g_ and T_d-5%_ of 171.3 °C and 341.7 °C, respectively.

## Figures and Tables

**Table 1:** Kinetic parameters and their correlation coefficients for the adsorption of formic acid.

Impurities	Pseudo-First-Order Model			Pseudo-Second-Order Model		
	q_e_ (mg/g)	k_1_ (min^−1^)	R^2^	q_e_ (mg/g)	k_2_ (g/mg/min)	R^2^
Formic Acid	5.92	0.112	0.983	6.42	0.316	0.992

**Table 2:** Moving boundary model parameters and their correlation coefficients for the adsorption of formic acid.

Impurities	Film Diffusion		Particle Diffusion		Chemical Reaction	
	R^2^	k_1_ (min^−1^)	R^2^	k_2_ (min^−1^)	R^2^	k_3_ (min^−1^)
Formic Acid	0.926	0.006	0.828	0.008	0.890	0.006

**Table 3:** Molecular weight and molecular structure of PIC synthesized from ISB with different properties.

ISB Sample ^a^	pH of ISB	Content of Formic Acid (mg/L)	M_n_ (kg/mol)	M_w_ (kg/mol)	PDI	Terminal Groups ^b^			Carbonyl Structures		
						endo-OH	exo-OH	-OPh	a_1_	a_3_	a_1_/a_3_
crude ISB	2.75	1.40	10.1	17.9	1.77	0.26	0.18	0.53	0.40	0.42	0.95
ISB-R1	7.48	0.41	48.9	80.2	1.64	0.15	0.09	0.32	0.47	0.45	1.04
ISB-R2	7.57	0.38	52.5	84.0	1.60	0.10	0.08	0.24	0.48	0.46	1.04
ISB-R3	7.22	0.49	41.6	69.5	1.67	0.18	0.09	0.37	0.46	0.45	1.02
ISB-R5	6.70	0.62	35.2	58.8	1.67	0.21	0.12	0.40	0.47	0.47	1.00
ISB-R10	5.53	0.83	27.3	46.9	1.72	0.23	0.14	0.48	0.43	0.45	0.96
ISB-T35	7.64	0.34	53.7	84.8	1.58	0.07	0.05	0.20	0.50	0.47	1.06
ISB-T45	7.68	0.34	53.9	85.2	1.58	0.07	0.05	0.20	0.50	0.47	1.06

^a^ Here, ISB-R1, ISB-R2, ISB-R3, ISB-R5, and ISB-R10 are the ISB purified at the flow rate of 1, 2, 3, 5, and 10 mL/min, respectively. And ISB-T35 and ISB-T45 are the ISB purified at 35 °C and 45 °C, respectively. ^b^ The content of terminal -OH and -OPh groups is calculated from ^1^H NMR by integrating the respective signals with MestReNova software (Version 11.0.4), using the backbone signal at δ 4.89 ppm as an internal reference, and assuming that this reference peak is unaffected by terminal groups and that all relevant protons have similar relaxation behavior.

**Table 4:** Physical properties of PIC synthesized from ISB with different properties.

ISB Sample	pH of ISB	Content of Formic Acid (mg/L)	T_g_ (°C)	T_d-5%_ (°C)	550 nm Transmittance (%)	Elongation at Break (%)	Young’s Modulus (GPa)
crude ISB	2.75	1.40	140.5	326.9	48.7	3.5	1.11
ISB-R1	7.48	0.41	168.4	336.9	85.5	7.1	1.70
ISB-R2	7.57	0.38	170.7	340.1	88.1	7.6	1.76
ISB-R3	7.22	0.49	168.0	334.4	82.5	5.7	1.54
ISB-R5	6.70	0.62	165.6	330.1	82.0	5.3	1.43
ISB-R10	5.53	0.83	158.3	327.3	80.9	5.2	1.16
ISB-T35	7.64	0.34	170.9	341.5	86.0	8.4	1.79
ISB-T45	7.68	0.34	171.3	341.7	84.7	10.2	1.84

## Data Availability

The original contributions presented in this study are included in the article. Further inquiries can be directed to the corresponding author.
